# Impact of Leptin on Periodontal Ligament Fibroblasts during Mechanical Strain

**DOI:** 10.3390/ijms22136847

**Published:** 2021-06-25

**Authors:** Agnes Schröder, Andrea Meyer, Gerrit Spanier, Anna Damanaki, Eva Paddenberg, Peter Proff, Christian Kirschneck

**Affiliations:** 1 Department of Orthodontics, University Hospital Regensburg, 93053 Regensburg, Germany; andrea.meyer@stud.uni-regensburg.de (A.M.); eva.paddenberg@ukr.de (E.P.); peter.proff@ukr.de (P.P.); christian.kirschneck@ukr.de (C.K.); 2 Department of Cranio-Maxillo-Facial Surgery, University Hospital Regensburg, 93053 Regensburg, Germany; gerrit.spanier@ukr.de; 3 Department of Periodontology and Operative Dentistry, University Medical Center of the Johannes Gutenberg University, 55131 Mainz, Germany; anna.damanaki@unimedizin-mainz.de

**Keywords:** periodontal ligament fibroblast, orthodontic tooth movement, leptin, mechanical strain

## Abstract

Orthodontic treatment to correct dental malocclusions leads to the formation of pressure zones in the periodontal ligament resulting in a sterile inflammatory reaction, which is mediated by periodontal ligament fibroblasts (PDLF). Leptin levels are elevated in obesity and chronic inflammatory responses. In view of the increasing number of orthodontic patients with these conditions, insights into effects on orthodontic treatment are of distinct clinical relevance. A possible influence of leptin on the expression profile of PDLF during simulated orthodontic mechanical strain, however, has not yet been investigated. In this study, PDLF were exposed to mechanical strain with or without different leptin concentrations. The gene and protein expression of proinflammatory and bone-remodelling factors were analysed with RT-qPCR, Western-blot and ELISA. The functional analysis of PDLF-induced osteoclastogenesis was analysed by TRAP (tartrate-resistant acid phosphatase) staining in coculture with human macrophages. Pressure-induced increase of proinflammatory factors was additionally elevated with leptin treatment. PDLF significantly increased RANKL (receptor activator of NF-kB ligand) expression after compression, while osteoprotegerin was downregulated. An additional leptin effect was demonstrated for RANKL as well as for subsequent osteoclastogenesis in coculture after TRAP staining. Our results suggest that increased leptin concentrations, as present in obese patients, may influence orthodontic tooth movement. In particular, the increased expression of proinflammatory factors and RANKL as well as increased osteoclastogenesis can be assumed to accelerate bone resorption and thus the velocity of orthodontic tooth movement in the orthodontic treatment of obese patients.

## 1. Introduction

The exact mechanisms of orthodontic tooth movement to therapeutically correct misaligned teeth and malocclusions with all their complex interrelationships have been studied for over 100 years [1] and are still an integral part of current research. Cellularly, apart from macrophages [2], T cells [3] and other cell populations, periodontal ligament fibroblasts (PDLF) comprise the major cell populations of the periodontal ligament and are subjected to compressive and tensile mechanical strain during orthodontic treatment [4]. These cells are responsible for the formation of collagen fibres, the regulation of tissue homeostasis and the activation of the non-specific immune system [5]. In response to compressive forces applied to correct tooth position, PDLF increasingly secrete proinflammatory enzymes, cytokines and chemokines [5,6,7,8]. In addition, the expression of prostaglandin-endoperoxide synthase 2 (PTGS2) increases in pressure zones, leading to an increase in leukotrienes and prostaglandin E2 [6]. Furthermore, through an autocrine feedback mechanism of PDLF, the expression of proinflammatory cytokines, such as interleukin-1 (IL1), interleukin-6 (IL6) and tumour necrosis factor (TNF), is increased [5,7,8]. In parallel, bone resorption is initiated in pressure zones. PTGS2 again plays a key role in this context. Prostaglandin E2 stimulates PDLF to a higher expression of soluble and membrane-bound RANKL (receptor activator of NF- kB ligand) [6]. RANKL binds to RANK on osteoclast progenitor cells, allowing them to fuse and differentiate into osteoclasts. This reaction is inhibited by osteoprotegerin (OPG). OPG is secreted by osteoblasts residing in the periodontal ligament, but also by PDLF. It is also able to bind to RANKL and thus inhibits osteoclastogenesis [9]. Previous studies have shown that the RANKL/OPG ratio shifts to the side of RANKL during orthodontic tooth movement in pressure zones, while inverse effects are observed in traction zones [5,8]. Systemic and exogenous factors can affect orthodontic tooth movement.

Obesity, as a possible result of poor diet and lack of exercise, is a growing problem in contemporary industrial societies [10]. Despite the high clinical relevance of this problem, there is not yet enough data to assess the impact of obesity on orthodontic tooth movement [11]. It is known that obesity negatively affects the entire organism due to favouring numerous diseases. Accordingly, weight gain and growing abdominal girth are associated with an increased risk of suffering from cardiovascular diseases, cancer, diabetes, asthma or osteoarthritis [12]. The relationship between obesity and periodontitis, an inflammatory, destructive disease of the periodontium, has been studied in depth. It has been shown that the risk of developing periodontitis increases with an increase in BMI, and that the risk of developing an increase in BMI increases with periodontitis [13,14,15]. Obesity is now considered the second-most important factor after smoking for inflammation-related periodontal bone loss [16]. The most common hypothesis explains the above diseases with the increased secretion of adipokines by adipocytes [11]. Adipokines are endocrine proteins and have pro-inflammatory, but also inhibitory properties linking metabolism to the immune system [17]. With weight gain, the pro-inflammatory adipokines are increasingly expressed [17]. It can thus be assumed that adipose tissue influences numerous struc-tures in the body through the regulation of inflammatory processes [18,19]. Leptin was the first adipokine to be discovered in 1994 [20]. Elevated leptin concentrations are also often associated with chronic inflammatory reactions [21]. In this context, an increase in proinflammatory cytokines through leptin has been demonstrated [22]. In addition, leptin directly influences regulatory T cells, which suppress the activation of the immune system and thereby regulate its self- tolerance [23]. Leptin can thus establish a link between nutritional status and the immune system [22]. The influence of leptin on bone metabolism has also been investigated in numerous studies. Leptin appears to be involved in both bone formation and breakdown [24,25,26,27,28]. Bone remodelling is essential for orthodontic tooth movement. With this in mind, the concentration of leptin in the sulcus fluid during orthodontic treatment has already been investigated [29,30]. An increase in leptin concentration was observed during the first phase of tooth movement. This was followed by a decrease, which ended after one month below the initial concentration. Furthermore, a clear positive correlation was observed between leptin concentration in the sulcus fluid and speed of tooth movement [30]. This could be one reason why faster tooth movement is observed in overweight children and adolescents [31]. The underlying cellular mechanisms and the impact of leptine on the sterile inflammatory reaction within the periodontal ligament enabling orthodontic tooth movement, however, remain unclear.

Understanding leptin and its influence on orthodontic tooth movement could make an important contribution to the long-term success of orthodontic treatment, especially in view of the increasing obesity rates in children and adolescents. The present in vitro study is thus intended to further elucidate the signalling cascades that occur during orthodontic treatment in obese patients focusing on fibroblasts of the periodontal ligament, which regulate orthodontic tooth movement, and to what extent leptin influences their metabolism.

## 2. Results

### 2.1. Expression of Leptin Receptors in PDLF and Effects of Different Leptin Concentrations

**Figure 1:**
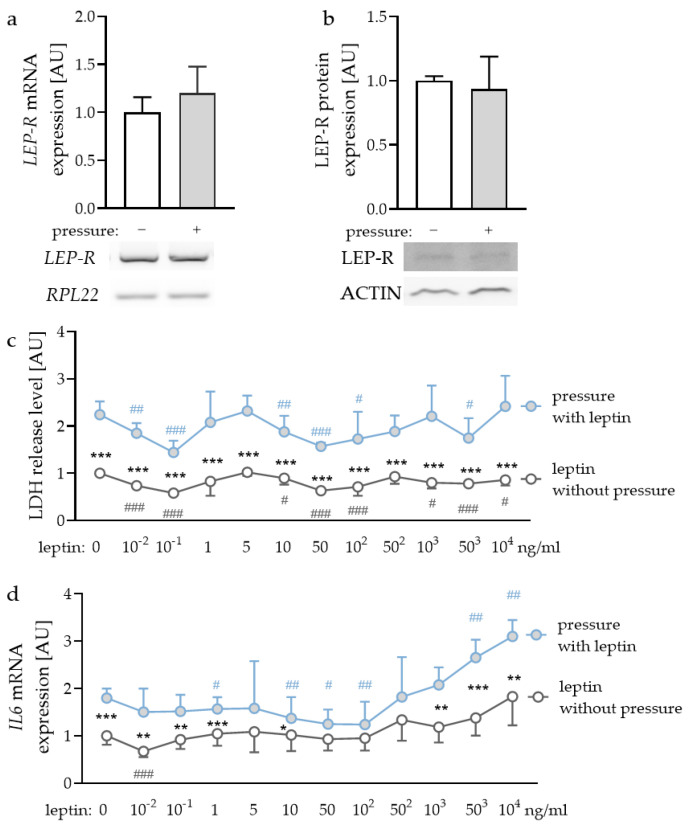
Gene (**a**) and protein expression (**b**) of the leptin receptor (LEP-R) in PDLF without and with compressive strain compared to the loading controls ribosomal protein L22 (*RPL22*) or ACTIN. Impact of different leptin concentrations without and with compressive strain on lactate dehydrogenase (LDH) release (**c**) and interleukin-6 (*IL6)* gene expression (**d**). n ≥ 6; Statistics: (**a**,**b**): Mann-Whitney U test; (**c**,**d**): ANOVA followed by unpaired *t* tests. * Pressure effect: comparison between control and pressure at the respective leptin concentration; * *p* < 0.05; ** *p* < 0.01; *** *p* < 0.001. ^#^ Leptin effect: comparison between respective leptin concentration and control or pressure without leptin addition. ^#^ *p* < 0.05; ^##^ *p* < 0.01; ^###^ *p* < 0.001.

First, we checked whether leptin receptors (LEP-R) were expressed in PDLF and if this expression was affected by compressive strain. We detected gene and protein expression of LEP-R in PDLF (Figure 1a,b), however, compressive strain did not affect gene (*p* = 0.240) or protein expression (*p* = 0.621). To determine the optimal leptin concentrations for the following experiments, we tested different concentrations ranging from leptin levels detectable in gingival fluid [32] and concentrations already reported to be effective for experiments with gingival fibroblasts and PDLF [33]. First, we checked for possible cytotoxic effects of the tested leptin concentrations. Compressive strain increased lactate dehydrogenase (LDH) release with all the tested leptin concentrations (Figure 1c). No investigated leptin concentration increased LDH release in PDLF, indicating that leptin had no cytotoxic effect (Figure 1c). The investigation of gene expression of interleukin-6 (*IL6*) revealed increased expression with compressive strain under control conditions (*p* < 0.001) and with low leptin concentrations (10^−2^ ng/mL: *p* = 0.008; 10^−1^ ng/mL: *p* = 0.007; 1 ng/mL: *p* < 0.001; 10 ng/mL: *p* = 0.042) and high leptin concentrations (10^3^ ng/mL: *p* = 0.001; 50^3^ ng/mL: *p* < 0.001; 10^4^ ng/mL: *p* = 0.002; Figure 1d). This effect was truncated with 50 ng/mL (*p* = 0.097); 10^2^ ng/mL (*p* = 0.087) and 50^2^ ng/mL leptin (*p* = 0.249). Leptin concentrations up to 50^3^ ng/mL increased *IL6* gene expression with compressive force treatment (50^3^ ng/mL: *p* = 0.002; 10^4^ ng/mL: *p* < 0.001; Figure 1d). As the concentration of 10^4^ ng/mL leptin showed a stable effect on *IL6* gene expression with no cytotoxic effects, we decided to use this concentration for the following experiments [33]. Furthermore, we tested 1 ng/mL, as this concentration can be detected in gingival fluid [32].

### 2.2. Impact of Leptin Combined with Compressive Strain on the Expression of Proinflammatory Factors

**Figure 2:**
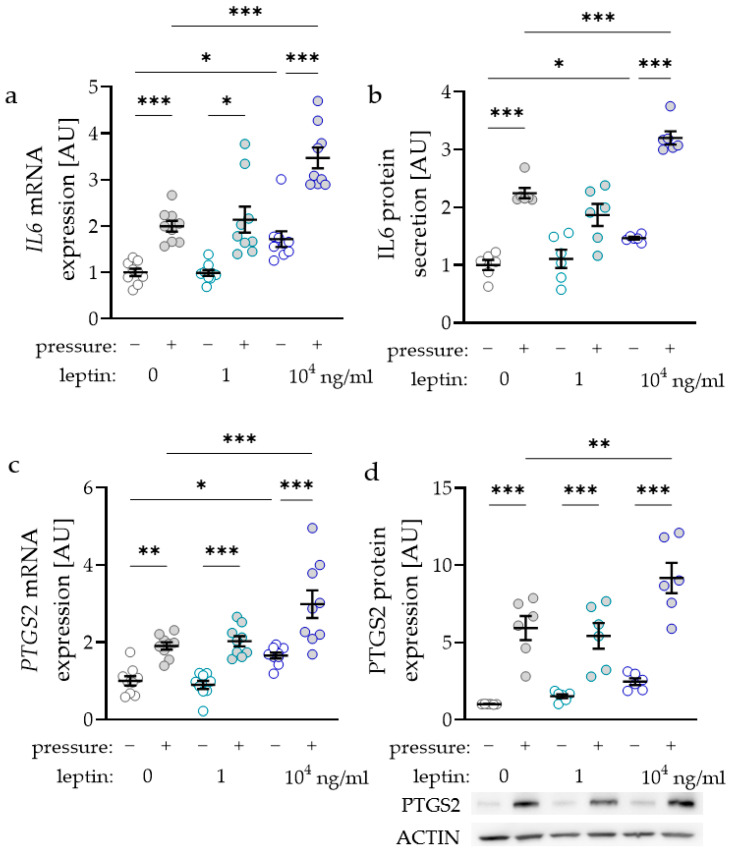
Impact of leptin in combination with compressive strain on gene and protein expression of interleukin-6 (IL6; **a**,**b**) and prostaglandin-endoperoxide synthase-2 (PTGS2; **c**,**d**) in PDLF; n ≥ 6; Statistics: ordinary ANOVA with Holm–Sidak’s multiple comparison tests (PTGS2) or Welch-corrected ANOVA with Games–Howell´s multiple comparison tests (IL6); * *p* < 0.05, ** *p* < 0.01, *** *p* < 0.001.

As the inflammatory factors interleukin-6 (IL6) and prostaglandin-endoperoxide synthase-2 (PTGS2) are known to modulate orthodontic tooth movement, we investigated the impact of leptin on the expression and secretion of these factors. *IL6* gene expression was elevated with compressive strain with all tested leptin concentrations (0 ng/mL: *p* < 0.001; 1 ng/mL: *p* = 0.029; 10^4^ ng/mL: *p* < 0.001; Figure 2a). Only treatment with the high leptin concentration of 10^4^ ng/mL further elevated *IL6* gene expression without (*p* = 0.026) and with compressive strain (*p* < 0.001), while the lower concentration of 1 ng/mL had no effect (Figure 2a). The results for *IL6* gene expression were reproducible at the protein level, as we detected more IL6 protein in cell culture supernatant with compressive force and with the high leptin concentration (Figure 2b). The gene expression of *PTGS2* was elevated with compressive strain with all tested leptin concentrations (0 ng/mL: *p* = 0.003; 1 ng/mL and 10^4^ ng/mL: *p* < 0.001; Figure 2c). Again, treatment with 10^4^ ng/mL leptin increased *PTGS2* gene expression without (*p* = 0.034) and with compressive strain (*p* < 0.001). In line with the RT-qPCR data, PTGS2 protein expression was upregulated with compressive strain with all tested leptin concentrations (*p* < 0.001; Figure 2d). Treatment with 10^4^ ng/mL leptin further enhanced pressure induced PTGS2 protein expression (*p* = 0.004).

### 2.3. Impact of Leptin Combined with Compressive Strain on the Expression of Bone-Remodelling Factors

**Figure 3:**
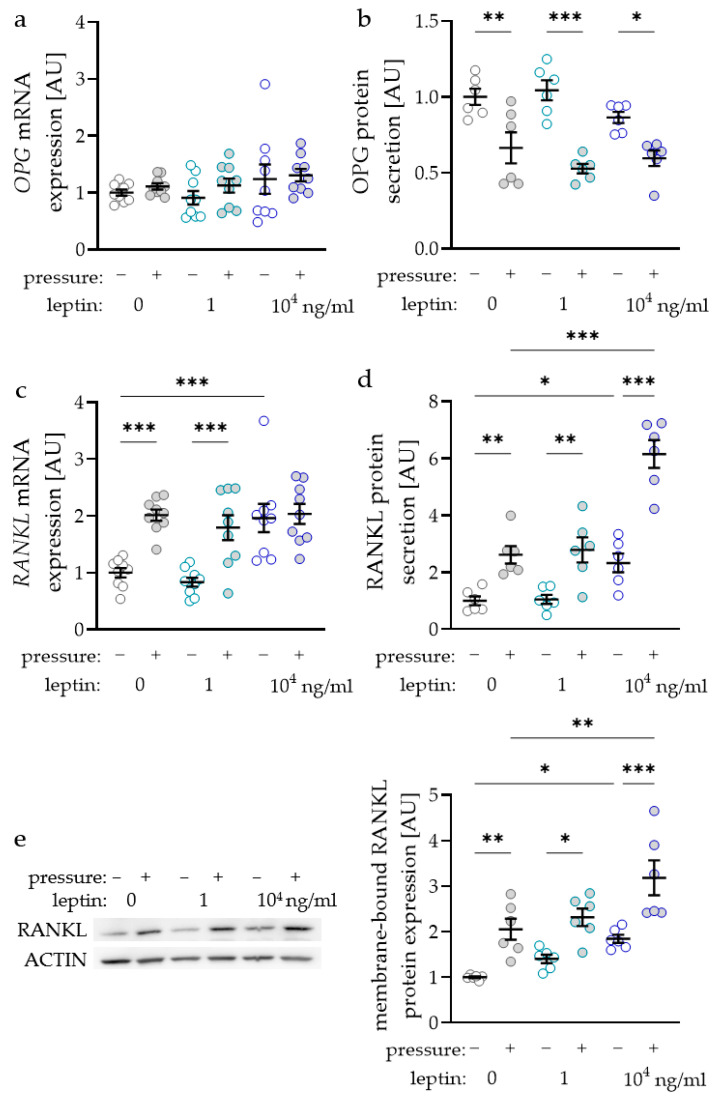
Impact of leptin in combination with compressive strain on gene and protein secretion of osteoprotegerin (OPG; **a**,**b**) and on gene and protein secretion respectively expression of membrane bound receptor activator of NF-kB ligand (RANKL; **c**–**e**) in PDLF; n ≥ 6; Statistics: ordinary ANOVA with Holm–Sidak’s multiple comparison tests; *OPG* mRNA: Welch-corrected ANOVA with Games–Howell’s multiple comparison tests; * *p* < 0.05, ** *p* < 0.01, *** *p* < 0.001.

The RANKL/OPG (receptor activator of NF-kB ligand/osteoprotegerin) system is essential for the differentiation of osteoclasts and therefore for alveolar bone remodelling during orthodontic tooth movement. Surprisingly, *OPG* gene expression was not affected by compressive strain with all tested leptin concentrations (0 ng/mL: *p* = 0.705; 1 ng/mL: *p* = 0.806; 10^4^ ng/mL: *p* > 0.999; Figure 3a). Treatment with leptin had no effect on *OPG* gene expression without or with compressive strain. Contrary to RNA data, we detected reduced OPG protein amounts in the cell culture supernatant after compressive force treatment with all tested leptin concentrations (0 ng/mL: *p* = 0.003; 1 ng/mL: *p* < 0.001; 10^4^ ng/mL: *p* = 0.021; Figure 3b). Again, leptin had no effect on OPG protein secretion, as we detected no differences without (1 ng/mL: *p* = 0.678; 10^4^ ng/mL: *p* = 0.410) and with compressive force (1 ng/mL: *p* = 0.410; 104 ng/mL: *p* = 0.678; Figure 3b). The gene expression of RANKL was elevated with compressive force without and with the low leptin concentration (*p* < 0.001; Figure 3c). With the higher leptin concentration *RANKL* gene expression was not elevated with compressive strain (*p* = 0.938), as it was already increased with leptin compared to the uncompressed control without leptin (*p* < 0.001). RANKL secretion in the cell culture supernatant was upregulated with compressive strain with all tested leptin concentrations (0 ng/mL: *p* = 0.003; 1 ng/mL: *p* < 0.001; 10^4^ ng/mL: *p* = 0.021; Figure 3b). The high leptin concentration increased RANKL secretion without (*p* = 0.028) and with compressive force (*p* < 0.001) compared to the control without leptin. Next to the RANKL secretion into the cell culture supernatant, we also investigated the expression of membrane bound RANKL via Western blot (Figure 3e). We observed increased RANKL expression after compressive strain with all tested leptin concentrations (0 ng/mL: *p* = 0.005; 1 ng/mL: *p* = 0.015; 10^4^ ng/mL: *p* < 0.001; Figure 3e). Again, the addition of 10^4^ ng/mL leptin increased RANKL expression without (*p* = 0.021) and with compressive strain (*p* = 0.003) compared to the control without leptin, indicating a positive effect of leptin on osteoclast differentiation.

### 2.4. Impact of Leptin Combined with Compressive Strain on Differentiation of Osteoclasts in a Coculture Model

**Figure 4:**
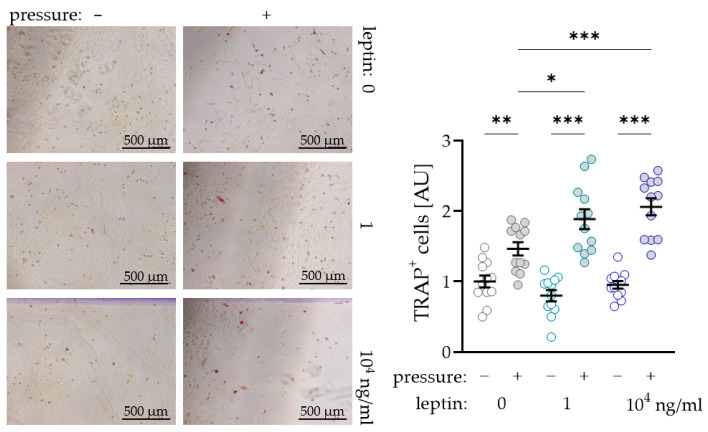
Impact of leptin in combination with compressive strain on the differentiation of osteoclast precursor cells to TRAP^+^ (tartrate-resistant acid phosphatase) osteoclasts via PDLF interaction; n ≥ 12; Statistics: ordinary ANOVA with Holm–Sidak´s multiple comparison tests; * *p* < 0.05, ** *p* < 0.01, *** *p* < 0.001.

To further investigate a possible supporting effect of leptin on osteoclast differentiation due to compressive strain in PDLF, we performed a coculture experiment with osteoclast precursor cells. As expected, we observed more TRAP^+^ (tartrate-resistant acid phosphatase) cells after compressive strain with all tested leptin concentrations (0 ng/mL: *p* = 0.007; 1 ng/mL and 10^4^ ng/mL: *p* < 0.001; Figure 4). Without compressive strain, leptin had no effect on osteoclastogenesis (1 ng/mL: *p* = 0.293; 10^4^ ng/mL: *p* = 0.733), while we detected more TRAP^+^ cells after compression with addition of the low (1 ng/mL: *p* = 0.012) and the high leptin concentration (10^4^ ng/mL: *p* < 0.001; Figure 4). These data support the findings for increased RANKL expression with unchanged OPG expression in PDLF under the influence of leptin.

## 3. Discussion

As obesity is a growing problem throughout society in all age groups, the dental specialty of orthodontics is also increasingly confronted with overweight patients [10]. It is already known that obesity has a negative effect on the entire organism due to numerous associated diseases [12]. An influence on the periodontal ligament linking teeth to their surrounding alveolar bone has already been shown in various studies. Next to smoking, obesity has been declared the most important risk factor for inflammatory processes in the periodontal ligament [16]. This is explained, among other things, by an increased secretion of endocrine-active proteins from adipose tissue, so-called adipokines [18]. An important adipokine that is responsible for the regulation of metabolism and additionally acts as a mediator in inflammatory reactions is leptin [34]. Elevated leptin concentrations are often associated with obesity or chronic diseases [35]. It is already known that leptin can contribute to an increased release of proinflammatory factors in PDLF and gingival fibroblasts, which also play an important role during orthodontic tooth movement [33]. However, there are hardly any data available so far in connection with orthodontic force application. Therefore, the present study aimed to investigate the possible impact of leptin on the expression profile of PDLF during simulated orthodontic mechanical strain.

Remodelling processes during orthodontic tooth movement are controlled, among other things, by the release of cytokines such as interleukin-6 (IL6) and the expression of prostaglandin endoperoxide synthase-2 (PTGS2). The compression of the PDLF evoked significantly increased expression of these inflammatory mediators, which was in line with previous studies [36,37,38]. After compression, increased IL6 expression at both gene and protein levels could be detected in both groups with leptin treatment. These results are consistent with those of previous studies that also observed increased expression of interleukins due to increased leptin concentration in experiments with PDLF [33]. After compression increased PTGS2 expression at both the gene and protein level was determined with addition of leptin. These results are supported by previous studies in other fields that linked increased PTGS2 activity to increased leptin concentrations [39]. In a larger, systemic context, leptin is associated with numerous chronic, non-autoimmune, and autoimmune diseases [40], which is consistent with the picture of an increased inflammatory response following leptin treatment in the present study.

The RANKL/OPG pathway is considered to be the central regulatory system of bone resorption [41]. RANKL (receptor activator of NF-kB ligand) promotes bone resorption by increasing the number and activity of functional osteoclasts [42]. This process can be inhibited by its soluble antagonist osteoprotegerin (OPG), which can be expressed by osteoblasts, but also PDLF in orthodontic context. Modulation of both RANKL and OPG expression can control bone resorption, which is essential for orthodontic tooth movement [43]. In their publication, Kanzaki et al. describe the connection between increased prostaglandin synthesis and the ratio of RANKL/OPG [6]. The balance of RANKL/OPG shifts to the RANKL side due to increased prostaglandin concentrations, leading to bone resorption at pressure zones of the periodontal ligament. Other studies demonstrated that increased prostaglandin levels stimulate bone resorption in fetal rats [44]. In our study, increased PTGS2 expression was accompanied by elevated RANKL expression due to pressure. OPG secretion was downregulated, which could also been shown in experiments with a similar experimental setup in the past [45,46,47,48]. A significantly increased RANKL expression was detected after compression with leptin treatment. These results were additionally confirmed by our analysis of osteoclastogenesis in coculture. We found a significant increase in osteoclast number in leptin-treated samples compared to untreated cells. These results seem to paint a clear picture of the relationship between increased leptin concentrations and bone resorption. Elevated leptin levels are often associated with obesity [49]. The present study, which revealed an increase in IL6 and PTGS2 and a concomitant increase in RANKL expression after leptin treatment, is thus in line with these previous findings. The effect of leptin on bone appears to be complex, as both positive [26] and negative influences [50] on bone density have already been reported in connection with leptin. Against this background, Cao and colleagues assume that the actual effect depends on the current leptin status and on central and peripheral effects [51]. With regard to orthodontic tooth movement, it is possible that the initial reaction at the beginning of treatment, which is characterised by an increased proinflammatory cytokine release at pressure zone, could be additionally elevated by high leptin concentrations [5].

Increased bone resorption and thus presumably increased orthodontic tooth movement, but also periodontal bone loss and dental root resorptions due to increased osteoclast activity may be a result of elevated leptin concentrations during orthodontic treatment of obese patients, which needs to be corroborated in further in vivo studies, especially in view of the increasing prevalence of obese patients in orthodontics. While increased tooth movement velocity in these patients would be favourable from a clinical perspective, the presumed risk for increased associated periodontal bone loss and dental root resorptions would suggest caution and diligence in orthodontic treatment of obese patients. Understanding the complex molecular processes triggered by adipokines such as leptin will hopefully help to better assess the risk for affected patients regarding unwanted side effects during orthodontic treatment and thus ensure successful therapy.

## 4. Materials and Methods

### 4.1. Cell Culture Experiments

Experiment 1: To identify the optimal leptin concentration for further analyses, pooled periodontal ligament fibroblasts (PDLF) from six different patients were seeded at a density of 70,000 cells per well on conventional polystyrene 6-well plates and preincubated with the leptin concentrations (cyt-228, Prospec, East Brunswick, NJ, USA) for 24 h under cell culture conditions in DMEM high glucose (D5671, Sigma-Aldrich, St. Louis, MO, USA) supplemented with 10% FBS (P30-3302, PAN-Biotech, Aidenbach, Germany), 1% antibioticum/antimycotic (A5955, Sigma-Aldrich, St. Louis, MO, USA) 1% L-Glutamine (G7513, Sigma-Aldrich, St. Louis, MO, USA), and 0.1% ascorbic acid (A8960, Sigma-Aldrich, St. Louis, MO, USA). The following leptin concentrations were tested: 0 ng/mL, 10^−2^ ng/mL, 10^−1^ ng/mL, 1 ng/mL, 5 ng/mL, 10 ng/mL, 50 ng/mL, 10^2^ ng/mL, 50^2^ ng/mL, 10^3^ ng/mL, 50^3^ ng/mL, 10^4^ ng/mL. Then, the cells either remained untreated (control) or were compressed using a sterile glass disc (2 g/cm^2^) for further 48 h, as already described and validated [6,37]. After the incubation period, mRNA was isolated and quantified by RT-qPCR. After evaluation of the results, it was decided to carry out the further experiments with 0 ng/mL (control), 1 ng/mL and 10^4^ ng/mL of leptin, respectively.

Experiment 2: To investigate the expression profile of PDLF under increasing leptin concentrations with simultaneous pressure application, 70,000 cells per well were seeded in conventional polystyrene 6-well plates (83.3920, Sarstedt, Nürnbrecht, Germany) and cultured for 24 h under cell culture conditions in full medium with different leptin concentrations. After 24 h of pre-incubation, cells remained untreated or were compressed for further 48 h using sterile glass discs (2 g/cm^2^) [6,37]. After 48 h of compression, gene and protein expression were examined by RT-qPCR, Western blots and ELISAs.

Experiment 3: For coculture experiments, PDLF were treated as described for experiment 2. After 48 h of compressive strain, 70,000 THP-1 cells (TIB-202; ATCC, Manassas, VA, USA) preincubated for 72 h with 50 ng/mL PMA (19-144, Sigma-Aldrich, St. Louis, MO, USA) in RPMI1640 (61871-044, Life Technologies, Carlsbad, CA, USA) and supplemented with 20% FBS (P30-3302, PAN-Biotech, Aidenbach, Germany), 1% antibiotic/antimycotic (A5955, Sigma-Aldrich, St. Louis, MO, USA) were added to the PDLF and incubated for another 72 h in RPMI1640 (61871-044, Life Technologies, Carlsbad, CA, USA) with 20% FBS (P30-3302, PAN-Biotech, Aidenbach, Germany) and 1% antibiotic/antimycotic (A5955, Sigma-Aldrich, St. Louis, MO, USA) under cell culture conditions. Afterwards, TRAP staining was performed to detect osteoclasts.

### 4.2. LDH Assay

A lactate dehydrogenase (LDH) test was performed according to the manufacturer’s instructions (04744926001, Roche, Penzberg, Germany) to test for possible cytotoxic effects of leptin. For this purpose, the same amount of cell culture supernatant was mixed with LDH solution consisting of catalyst and staining solution in a ratio of 1:45 in a 96-well plate (10-121-0000, nerbe plus, Winsen/Luhe, Germany) and incubated for 30 min in the dark at room temperature. After addition of stop solution, the absorbance at 490 nm and 690 nm was measured with an ELISA reader (Multiscan GO, Thermo Fisher Scientific, Waltham, MA, USA). Background absorbances at 690 nm were subtracted from the primary wavelength measurement at 490 nm.

### 4.3. RNA Isolation and cDNA Synthesis

RNA extraction was performed using the Trizol method. After the cells were scraped off in 1 mL PBS, they were centrifuged at 2000 rpm for 5 min at 4 °C (HERAEUS Fresco 17 Centrifuge, Thermo Fisher Scientific, Waltham, MA, USA). The supernatant was carefully removed and 500 µL RNA Solv (R6830-01, VWR international, Radnor, PA, USA) were added to the pellet, followed by 100 µL chloroform. The suspension was vortexed for 30 s and incubated on ice for 15 min. Samples were centrifuged at 13,000 rpm for 15 min at 4 °C. The aqueous supernatant was mixed with 500 µL isopropanol (20.842.3302, VWR international, Radnor, PA, USA) and stored at −80 °C overnight. After centrifugation at 13,000 rpm at 4 °C for 30 min the supernatant was discarded, and the pellet was washed in 500 µL 80% EtOH twice. The pellet was dried for at least 30 min, resuspended in 20 µL sterile, RNase-free water (T143.5, Carl Roth, Karlsruhe, Germany) and measured in the photometer (N60, Implen, Munich, Germany). The obtained RNA was transcribed to cDNA for the following semi-quantitative PCR and RT-qPCR. The same amounts of RNA (75 ng) were mixed with a previously prepared master mix consisting of 1x MMLV buffer (M1705, Promega, Madison, WI, USA), 0.1 nmol oligo-dT (SO131, Thermo Fisher Scientific, Waltham, MA, USA), 0.1 nmol random hexamer (SO142, Thermo Fisher Scientific, Waltham, MA, USA), 10 mM dNTPs (L785.2, Carl Roth, Karlsruhe, Germany), 40 U RNase inhibitor (EO0381, Thermo Fisher Scientific, Waltham, MA, USA) and 200 U reverse transcriptase (M1705, Promega, Madison, WI, USA) in a total volume of 10 µL. The samples were incubated for 1 h at 37 °C, followed by heat inactivation of the reverse transcriptase for 2 min at 95 °C. Afterwards, the samples were diluted addition of 40 µL sterile, RNase-free water (T143.5, Carl Roth, Karlsruhe, Germany).

### 4.4. Semiquantitative PCR and Agarose Gel Electrophoresis

For each sample, 2 µL of the obtained cDNA (corresponding to 3 ng of transcribed RNA) were mixed with 18 µL master mix consisting of 14.4 µL RNase free H_2_O_dd_ (T143.5, Carl Roth, Karlsruhe, Germany), 0.5 µL forward and reward primer (Table 1), 2 µL 10× buffer with MgCl2 (49150600, Roche, Penzberg, Germany), 0.4 µL dNTPs (14231323, Roche, Penzberg, Germany), and 0.2 µL Taq polymerase (51944300, Roche, Penzberg, Germany). cDNA was amplified (95 °C for 5 min, 40 cycles each 95 °C for 20 s; 60 °C for 1 min) and loaded on 1% of agarose gel (6351.5, Carl Roth, Karlsruhe) with Gel Red (41003m, Biotum, Hayward, CA, USA) at 120 V for 40 min. Detection was carried out under UV light using the VWR Genoplex documentation system (VWR international, Radnor, PA, USA).

### 4.5. Quantitative Real-Time Polymerase Chain Reaction (RT-qPCR)

For RT-qPCR, 1.5 µL cDNA (corresponding to 2.25 ng of transcribed RNA) with 8.5 µL of a previously prepared primer mix was pipetted in duplicate into a 96-well plate (712282, Biozym, Hessisch Oldendorf, Germany), covered with an adhesive optical film (T12350, Biozym, Hessisch Oldendorf, Germany) and briefly centrifuged. The primer mix consisted of 0.25 µL forward and reward primer (Table 1), 3 µL RNase free H_2_O_dd_ (T143.5, Carl Roth, Karlsruhe, Germany) and 5 µL Luna Universal qPCR Master Mix (M3003E, New England Biolabs, Ipswich, MA, USA) per sample. The plates were put in the Realplex2 (Eppendorf, Hamburg, Germany) and run with the appropriate programme (95 °C for 2 min, 45 cycles each of 95 °C for 10 s, 60 °C for 20 s and 72 °C for 8 s). To determine relative gene expression, the formula 2^−ΔCq^ [52], with ∆C_q_ = C_q_ (target gene) − C_q_ (geometric mean *PPIB/RPL22* [53]) was used (Table 1).

### 4.6. Western Blot Analysis

Proteins were isolated with 100 µL CelLytic (C2978, Sigma-Aldrich, St. Louis, MO, USA) per well mixed with proteinase inhibitor (87786, Thermo Fisher Scientific, Waltham, MA, USA). Protein concentration was determined with RotiQuant (K015.2, Carl Roth, Karlsruhe, Germany). The same amounts of proteins for each sample were mixed with protein loading buffer and heated for 7 min at 70 °C. Proteins were separated on 10% separation gels for 90 min at 120 V. Proteins were then transferred to a polyvinylidene fluoride membrane (PVDF, T830.1, Carl Roth, Karlsruhe, Germany) in 90 min at 90 V using a tank blot. After blocking the membranes for 1 h at room temperature in 5% milk in TBS-T, membranes were incubated overnight in the primary antibody at a dilution of 1:1000 (LEP-R: PA1-053, Invitrogen, Waltham MA, USA; RANKL: TA306362, Origene, Rockville, MD, USA; PTGS2: PA5-16817, Thermo Fisher Scientific, Waltham, MA, USA; ACTIN: A2066, Sigma-Aldrich, St. Louis, MO, USA). After the membranes were washed three times for 5 min with TBS-T, they were incubated for 60 min with the secondary antibody (611-1302, Rockland Immunochemicals, Gilbertsville, PA, USA), which had been diluted 1:5000 in 5% milk in TBS-T, and washed again three times for 5 min each in TBS-T. Now the membranes were incubated with Luminata Crescendo Western HRP Substrate (WBLUR0100, Sigma-Aldrich, St. Louis, MO, USA) and signals could be detected using VWR Genoplex (VWR international, Radnor, PA, USA). Densitometric evaluation was done with the software ImageJ (National Institutes of Health, Bethesda, MD, USA). To test more than one protein expression the membrane was washed for 10 min at room temperature in TBS-T three times and incubated for 20 min in Re-Blot Plus Mild (10×) (2502, Merck, Kenilworth, NJ, USA). Before another primary antibody was used, the membrane was blocked for 1 h in 5% milk in TBS-T.

### 4.7. Enzyme-Linked Immunosorbent Assay (ELISA)

ELISAs were performed for the detection of RANKL (RD193004200R, Biovendor, Brno, Czech Republic), OPG (178092220, Thermo Fisher Scientific, Waltham, MA, USA) and IL6 (1311639521, Boster Bio, Pleasanton, CA, USA) according to the respective manufacturer’s instructions.

### 4.8. TRAP (Tartrate-Resistant Acid Phosphatase) Staining

The 6-well plates were removed from the incubator and the cells carefully washed with 500 µL PBS. The cells were fixed with 400 µL 10% glutaraldehyde solution. The glutaraldehyde solution was removed, and the cells were washed with 400 µL PBS at least three times. The cells were coated with 400 µL freshly prepared TRAP solution (0.3 mg Fast Red Violet LB stain (F-3381, Sigma-Aldrich, St. Louis, MO, USA) per mL TRAP Buffer (50 mL 0.1 M Acetate Buffer, 10 mL 0.3 M Sodium Tartrate, 1 mL 10 mg/mL Naphtol AS-MX Phosphate (N-5000, Sigma-Aldrich, St. Louis, MO, USA), 100 µL Triton X-100, 38.9 mL H_2_O_dd_) and incubated at 37 °C for 15 min. Again, the cells were washed twice with 400 µL PBS. The TRAP^+^-cells were quantified in 17 visual fields under the microscope (Olympus IX50).

### 4.9. Statistical Analysis

In Figure 1 bars or symbols represent mean values and the vertical lines show the standard deviation. In Figure 2, Figure 3 and Figure 4 symbols represent individual data points; horizontal lines show mean values; and vertical lines the standard error of the mean. We checked for normal distribution of the data performing a Shapiro-Wilk test. For comparison of two data sets (Figure 1a,b) Mann-Whitney tests were performed. For analysis of leptin concentration kinetics an ANOVA followed by an uncorrected multiple comparison test (two tailed unpaired *t*-test) was used. Depending on normal distribution of the data sets, either an ANOVA with a Holm–Sidak’s multiple comparison tests or a Welch-corrected ANOVA with Games–Howell´s multiple comparison tests was chosen. Differences were considered statistically significant at *p* < 0.05. Statistical analysis was performed with Graph Pad Prism Version 9.1 (GraphPad Software, San Diego, CA, USA).

## Figures and Tables

**Table 1:** Primer sequences for reference genes (*PPIB/RPL22*) and target genes.

Symbol	Gene Name	5′-Forward Primer-3′	5′-Reverse Primer-3′
*IL-6*	interleukin-6	TGGCAGAAAACAACCTGAACC	CCTCAAACTCCAAAAGACCAGTG
*LEP-R*	leptin receptor	CAGAAGCCAGAAACGTTTGAG	AGCCCTTGTTCTTCACCAGT
*OPG*	osteoprotegerin	TGTCTTTGGTCTCCTGCTAACTC	ACGCTCCAGGACTTATACCG
*PPIB*	peptidylprolyl isomeraseA	TTCCATCGTGTAATCAAGGACTTC	GCTCACCGTAGATGCTCTTTC
*PTGS2*	prostaglandin-endoperoxide synthase-2	GAGCAGGCAGATGAAATACCAGTC	TGTCACCATAGAGTGCTTCCAAC
*RANKL*	receptor activator of NFkB ligand	ATACCCTGATGAAAGGAGGA	GGGGCTCAATCTATATCTCG
*RPL22*	ribosomal protein L22	TGATTGCACCCACCCTGTAG	GGTTCCCAGCTTTTCCGTTC

## Data Availability

All datasets are publically available upon request from the corresponding author.
